# 3D-Braided Poly-ε-Caprolactone-Based Scaffolds for Ligament Tissue Engineering

**DOI:** 10.3390/jfb13040230

**Published:** 2022-11-08

**Authors:** Caroline Emonts, David Wienen, Benedict Bauer, Akram Idrissi, Thomas Gries

**Affiliations:** Institut für Textiltechnik (ITA), RWTH Aachen University, 52074 Aachen, Germany

**Keywords:** 3D braiding, textile scaffold, poly-ε-caprolactone (PCL), anterior cruciate ligament (ACL), ligament repair, tissue engineering

## Abstract

The anterior cruciate ligament (ACL) is the most commonly injured intra-articular ligament of the knee. Due to its limited intrinsical healing potential and vascularization, injuries of the ACL do not heal satisfactorily, and surgical intervention is usually required. The limitations of existing reconstructive grafts and autologous transplants have prompted interest in tissue-engineered solutions. A tissue engineering scaffold for ACL reconstruction must be able to mimic the mechanical properties of the native ligament, provide sufficient porosity to promote cell growth of the neoligament tissue, and be biodegradable. This study investigates long-term biodegradable poly-ε-caprolactone (PCL)-based scaffolds for ACL replacement using the 3D hexagonal braiding technique. The scaffolds were characterized mechanically as well as morphologically. All scaffolds, regardless of their braid geometry, achieved the maximum tensile load of the native ACL. The diameter of all scaffolds was lower than that of the native ligament, making the scaffolds implantable with established surgical methods. The 3D hexagonal braiding technique offers a high degree of geometrical freedom and, thus, the possibility to develop novel scaffold architectures. Based on the findings of this study, the 3D-braided PCL-based scaffolds studied were found to be a promising construct for tissue engineering of the anterior cruciate ligament.

## 1. Introduction

The anterior and posterior cruciate ligaments are essential components for stabilizing the knee joint in rotational motion and securing the tibia in the anterior-posterior direction. At the same time, due to its structure, the anterior cruciate ligament (ACL) represents a major weak point, as its rupture is the most frequent injury in the knee joint, accounting for 46% of cases. Due to the complex demands on the cruciate ligament, it is mostly replaced with an autologous transplant in case of an ACL rupture [1].

Although autologous transplants show functional clinical results, they have numerous disadvantages, including limited availability, impairment of secondary knee stability, possible donor site complications, and longer surgical duration due to graft preparation [2,3,4,5].

Commercial permanent synthetic ligaments are an alternative. These show suitable mechanical and functional results in the short term after surgery. Long-term clinical outcomes are limited due to mechanical disparity, lower abrasion resistance of synthetic ligaments, and limited integration between the graft and host tissue [6,7,8].

Tissue engineering offers a new reconstruction and treatment method for ACL ruptures. The reconstruction of the ligament tissue can be supported by mechanical and morphological optimization of the scaffold.

The mechanical properties of the ACL native serve as a guideline for the scaffold development, these are dependent on the patient’s age, sex, and level of sport activity. The tensile strength of the ACL ranges from 734 to 2160 N, with an elongation of 37%. The load on the ACL in daily life varies between 169 N (walking) and 445 N (descending stairs). The stiffness of the ACL ranges from 75 to 242 N/mm [9,10,11,12,13]. The minimum cross-sectional area is given with 44 mm^2^ [10].

Previous studies in the field of tendon and ligament tissue engineering with degradable scaffolds can be divided into two groups concerning the material. The first deals with natural polymers such as silk or collagen. Natural polymers offer the advantages of suitable biocompatibility and biofunctionality. However, there are also limitations to the manufacturing process, as it is rarely scalable, and there is a large batch dependence that limits reproducibility. Furthermore, the mechanical properties of the fibers of natural polymers, except silk, are not suitable for load-bearing tissues such as tendons or ligaments. Silk has the disadvantage that the raw material, as well as the processing, are very expensive, and by the removal of the immunogenic sericin protein, the biomechanical properties are altered. [14,15,16]

Synthetic biodegradable polyesters such as polylactic acid (PLA), polyglycolic acid (PGA) and their copolymer polylactic-co-glycolic acid (PLGA) offer a stable, reproducible process and nearly unrestricted availability. During degradation, polylactide-based polymers give rise to acidic degradation products. In poorly vascularized tissues such as ligaments, this can lead to inflammatory reactions [17,18,19]. In addition, in other studies, a tendency for the insufficient long-term stability of polylactide materials is shown [20,21,22]. Regeneration of the ACL and its ensuing functionality usually require at least 6 months [23]. In these applications, slower degrading materials should be selected [23,24,25].

Due to the limitations of the previously mentioned materials in terms of mechanical properties, reproducibility, and acidic degradation products, poly-ε-caprolactone (PCL) was used in the present study. PCL is an aliphatic polyester with slow degradation. Despite its acidic degradation product, ε-hydroxy caproic acid, PCL is considered to only have a negligible tendency to cause an acidic environment. [26,27,28] In long-term in vitro hydrolysis studies, no or only minimal changes in pH were observed [29,30]. Lam, Hutmacher et al. have studied the biocompatibility of PCL during in vivo degradation reporting no adverse effects [28,31]. In tissue engineering, PCL has been widely used in the form of nanofibers, however, these are, due to their strength, not suitable for the replacement of human load-bearing structures [28,32]. The use of melt-spun PCL fibers in tissue engineering has rarely been reported [33,34]. We have presented promising results on the fabrication of melt-spun PCL fibers with different cross-section geometries for tissue engineering applications [35]. Similar fibers were used in the present study.

Textile manufacturing methods offer great variability for producing tissue engineering scaffolds. Textile scaffolds can provide a 3D structure with reproducible and scalable manufacturing [36]. In addition, textiles can be designed to be load-efficient and capable of high strength. In the case of tensile-loaded structures such as tendons and ligaments, braids are particularly suitable for scaffold production [37]. In braids, all fibers are mostly oriented in the direction of the tensile load. Furthermore, the mechanical properties are adjustable within a wide range via the braiding parameters number of filaments, braiding angle, braiding pattern, and braiding geometry [38,39].

Besides the mechanical properties, the morphological properties such as pore size and porosity, as well as the diameter and width of the scaffold can be modified by the braiding parameters [40,41]. Another advantage of braided scaffolds is the structural similarity to the hierarchical structure of the native ligament of fibers and fibrils. This biomimetic structure can be useful for the reconstruction of native tissue [37,42].

The biomimetic approach to designing tissue-engineered scaffolds for ACL replacement was also followed by Cooper et al. [43]. Using polylactide-co-glycolide 10:90 (PLAGA) fibers, 3D circular braiding and rectangular braiding methods were evaluated regarding their suitability for scaffold production. The scaffold was designed in particular to meet the various porosity and pore size requirements of bone and ligament tissue. The 3D circular fibrous scaffold was able to withstand tensile loads of 907 N ± 132 N. The study of Lu et al. showed the biological suitability of the aforementioned 3D-braided scaffolds [44]. Mengsteab et al. used a track and column bench-top braiding machine to fabricate 3D-braided PLLA scaffolds. The scaffolds were characterized regarding mechanical properties as a function of braiding height. A scalability up to 2494 ± 59.63 N at a cross-sectional area of 12.57 mm^2^ was shown [45].

Conventional circular braiding has been used in several studies to produce biodegradable scaffolds for ACL replacement. Laurent et al. have simulated and validated the mechanical properties of poly(lactide-co-ε-caprolactone)-based (PLCL) ACL scaffolds from fiber to scaffold level [41]. Freeman et al. investigated the mechanical properties and cell response of braided, twisted, and braid-twisted poly(L-lactic acid) (PLLA) fibers [46]. These studies show the potential of braiding technology for the production of tissue-engineered ligaments.

The 3D hexagonal braiding technology offers advantages for processing sensitive and degradable materials. The high packing density of the bobbins in the machine bed minimizes the yarn drop during the braiding process. This in turn minimizes friction during the braiding process and facilitates the processing of sensitive degradable polymer fibers. This 3D braiding technology offers large freedom of shape for the geometry of the scaffold so that pore size and porosity can be adjusted [47,48,49]. Moreover, the 3D braids produced are interconnected in their different layers. This allows the creation of guiding structures for three-dimensional cell ingrowth.

Our overall approach to the design of functional ACL scaffolds was focused on several parameters: polymer material, scaffold shape, mechanical properties, porosity, and pore size, as well as degradability [35]. In this paper, the particular focus is on understanding the effects of varying the parameters of 3D-braiding geometry and braiding position on the mechanical properties of the fabricated scaffold. For this purpose, seven geometries of 3D hexagonal braids are developed and characterized. These are distinguished by the load-bearing orientation of the fibers and struts in all three spatial directions, resulting in three-dimensional guide structures for future cell seeding.

## 2. Materials and Methods

### 2.1. Scaffold Production

**Figure 1:**
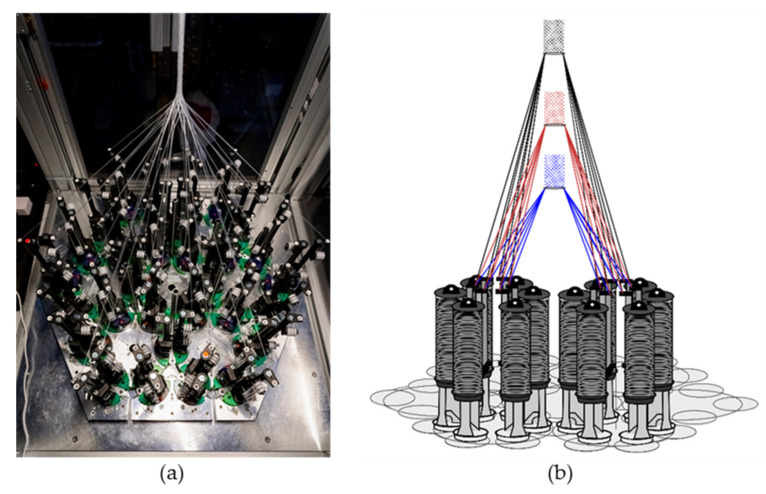
(**a**) Hexagonal braiding machine at Institut für Textiltechnik of RWTH Aachen University, Germany, and (**b**) scheme of the machine bed with different braiding positions (blue = position 1, red = position 2, black = position 3).

The hexagonal braiding machine of the Institut für Textiltechnik of RWTH Aachen University, Germany (Figure 1a) was used to produce the braided scaffolds. On the braiding machine, 36 fine wire bobbins (Körting Nachfolger Wilhelm Steeger GmbH & Co. KG, Wuppertal, Germany) with 3 deflections were mounted. The used thread tension was 0.33 N. The braids were made of PCL monofilaments with a round cross-section. The PCL monofilaments are manufactured at Institut für Textiltechnik of RWTH Aachen University, Germany. PCL-pellets with a molecular weight of 80,000 Da (CAPA 6800^®^, Perstorp Holding AB, Malmö, Sweden) were used for the melt-spinning process described in [35]. Seven monofilaments were folded per bobbin to achieve the mechanical properties of the ACL in a single braiding step. Each braid consisted of 252 filaments in total. The fiber properties are listed in Table 1.

In the present study, seven different geometries were investigated. Each of these geometries was braided at three braiding positions. These correspond to different braiding angles in conventional braiding processes. The braiding positions are defined by the distance between the bobbin and the braiding point of 17 cm (position 1), 25 cm (position 2), and 33 cm (position 3), respectively (Figure 1b). Through this design of experiment, the influences of braiding geometry and braiding position were investigated.

### 2.2. Braiding Geometries

**Figure 2:**
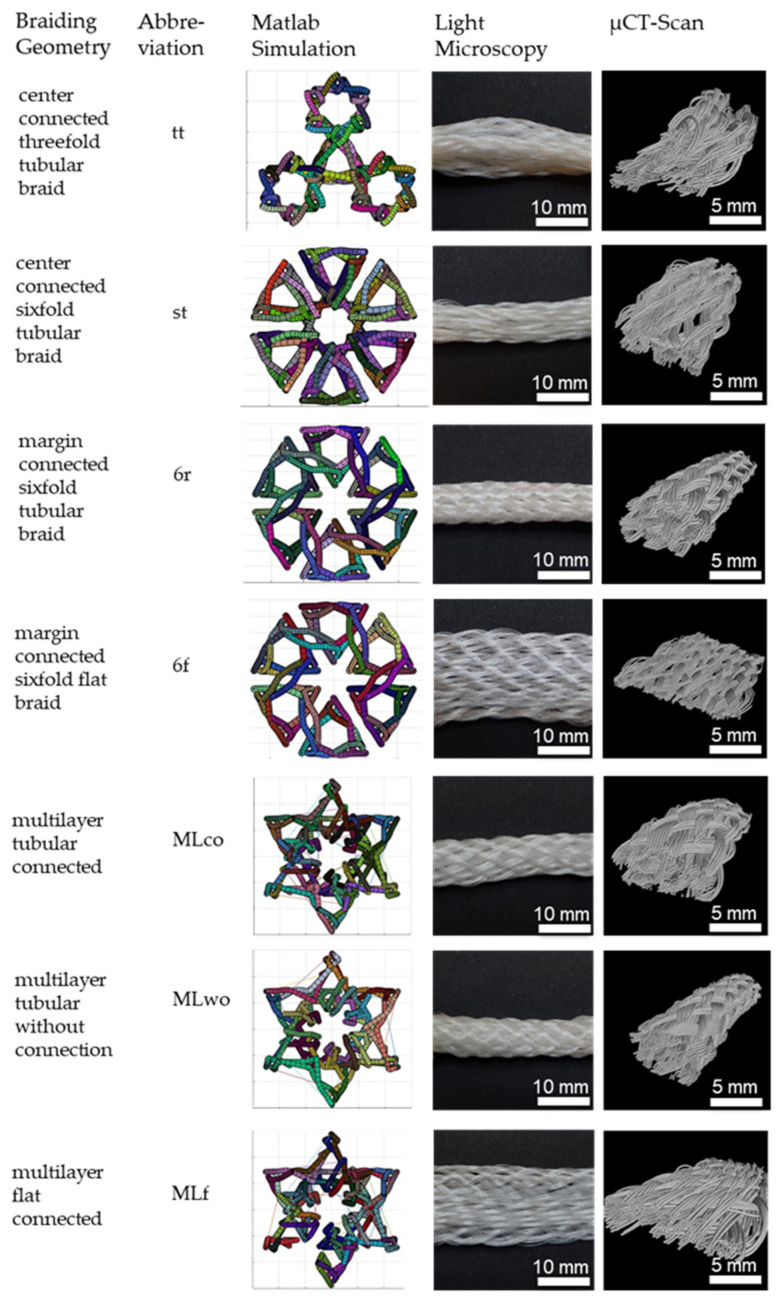
3D-braided PCL-based scaffolds, shown is the cross-section of the Matlab visualization, the top view using light microscopy, and µCT-scan. Light microscopy pictures and µCT scans depict braids manufactured in position 1.

Seven different 3D braids are investigated in this study. 3D braids are suitable for scaffold application due to their filament orientation in all three spatial directions to provide a 3D guiding structure for cell growth. The developed braids are shown in Figure 2.

The braided scaffolds can be divided into arranged tubular braids and multilayer braids. Whereby both groups can be realized as braids with a round or a flat cross-section. The tubular braids were a combination of three or six tubes connected through the center or the margin. The “center connected threefold tubular braid” (tt) is inspired by the physiological structure of the anterior cruciate ligament with its three bundles. A scaling of the tt braid is the “center connected sixfold tubular braid” (st). The variation of the connection position leads to the “margin connected sixfold tubular braid (6r)” and the “margin connected sixfold flat braid (6f)”. The group of multilayer braids contains braids with two layers in one another. The layers can be connected or unconnected. The group contains the “multilayer tubular connected” (MLco), “multilayer tubular without connection” (MLw/o), and the “multilayer flat connected” (MLf).

The braids were compared in terms of the connection across the center or margin, the influence of flat or round arrangement, and the influence of the connection in multilayer braids. The comparison of the different braiding geometries is shown in Table 2.

### 2.3. Matlab Visualization

The different braiding geometries were designed based on the physiological structures of the ligament and possibilities in freedom of shape using the hexagonal braiding machine. Visualization of the different braids was achieved due to a Matlab program developed in preliminary work. The program calculates the geometry of the braid based on the braiding paths specified by the user. The developed yarn carrier path was manually transferred to the machine interface [48].

### 2.4. Tensile Tests

**Figure 3:**
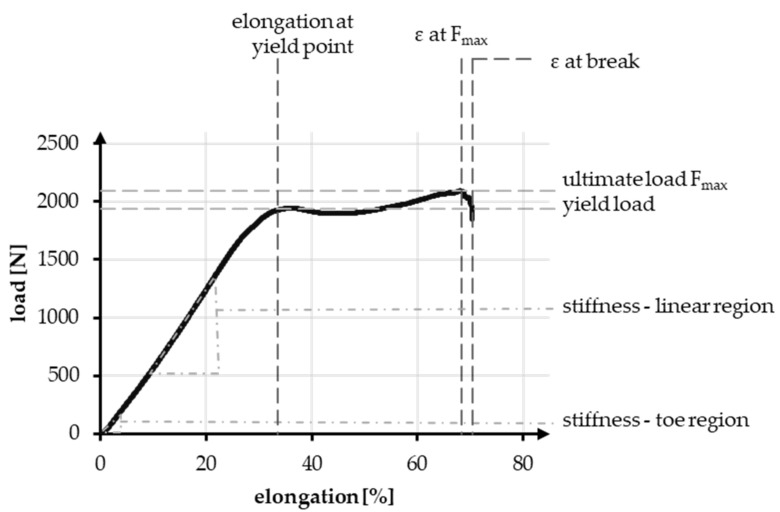
Exemplary tensile test curve of a PCL-based 3D-braided ACL scaffold, including the measured values and defined regions.

Tensile tests in accordance with standard NF S 94-167-2 were performed. The tests were carried out using the universal testing machine ZmartPro, ZwickRoell GmbH & Co. KG, Ulm, Germany. Before testing, all specimens were equipped with cardboard force application elements using araldite resin. According to DIN EN ISO 139, all specimens were conditioned in standard climate for 24 h before testing. A gauge length of 100 mm and 40 mm was used. The test speed was adjusted to 100% of the specimen length per minute, which led to 100 mm/min and 40 mm/min, respectively. All specimens were tested with a pre-load of 2 N in accordance with NF S 94-167-2. A number of 5 samples was used for each experiment. The characteristic values examined during the tensile test are presented in Figure 3, comparable to Korhonen et al. [50]. Included in the values are elongation, load, and stiffness. The characteristic values in the linear region are compared to the physiological values of a native anterior cruciate ligament.

The linear region is limited by the yield load and the elongation at that load. The stiffness can be described with the gradient, similar to Young’s modulus and is defined as the absolute required load in N to achieve a displacement of 1 mm, normalized over the gauge length. During tensile tests, the stiffness in the linear region was determined between 200 and 1000 N, whereas the stiffness in the toe region is determined between 10 and 40 N. The absolute elongation stiffness is theoretically increased with the reduction of specimen length from 100 to 40 mm, by a factor of 2.5. The factor is verified in an experimental comparison.

### 2.5. Scaffold Morphology

**Figure 4:**
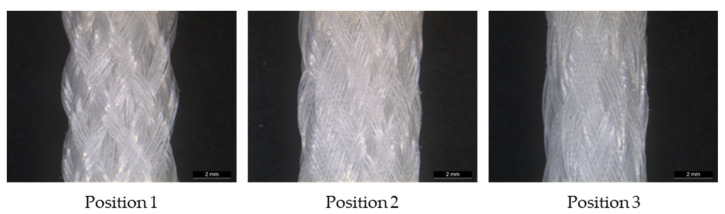
Light microscopy image of MLwo braid at the three braiding positions.

The 3D scaffolds were investigated using micro-computed tomography (µCT) scans to determine the porosity and pore size. The influence of the braiding position on the porosity is shown in Figure 4. One specimen of each shape and position was analyzed. For µCT scan processing the software GeoDict, math2Market GmbH, Germany was used. The pore diameter was calculated in µm by assuming spherical pores. The pores in the range of 0 to 500 µm were analyzed. The porosity is given in percentage and defined as the ratio between the cavity and the total volume of the scaffold.

An analysis of the cross-section provides information on the physiological implantability of the braid in the knee. Implantability is ensured if the cross-section of the braid is at most equal to that of the native cruciate ligament and does not exceed the usual surgical drill hole size of 7–9 mm [5]. The cross-section of the cruciate ligament can be assumed to be 44 mm^2^ [10], that of the drill hole 38.48–63.62 mm^2^ [9]. The dimensions of the braid are measured with a caliper gauge under a load of 2 N. This load corresponds to the pre-load applied in the tensile tests. One braid of each shape and position is measured. The different braiding shapes are averaged between the 3 positions.

### 2.6. Statistics

The analysis, presented in the graphs and tables is specified in the form of mean ± standard deviation. It was statistically examined using an unpaired t-test. In the case of multiple comparisons, two-factor analysis of variance (ANOVA) was conducted. The Tukey–Kramer test was used as a posthoc test, respectively. A significance level of 5% is applied in both cases. The statistical analysis is carried out using the software Microsoft Excel, Microsoft Corporation, United States of America.

## 3. Results

### 3.1. Mechanical Properties of the Scaffolds

The tensile tests were used to record and evaluate the mechanical values of the hexagonal braids. The quantitative data are listed in Table 3. Changing the braid shapes had a significant influence (*p* < 0.001) on the ultimate load of the braid, the elongation at the ultimate load, and the elongation at break. Changing the braid positions only influenced the elongations significantly (*p* < 0.001) but had no significant influence on the ultimate load (*p* = 0.15). A higher braid position resulted in a lower elongation. On average, the elongation was 73.53 ± 8.53%, at an average ultimate load of 2133.15 ± 113.07 N.

**Figure 5:**
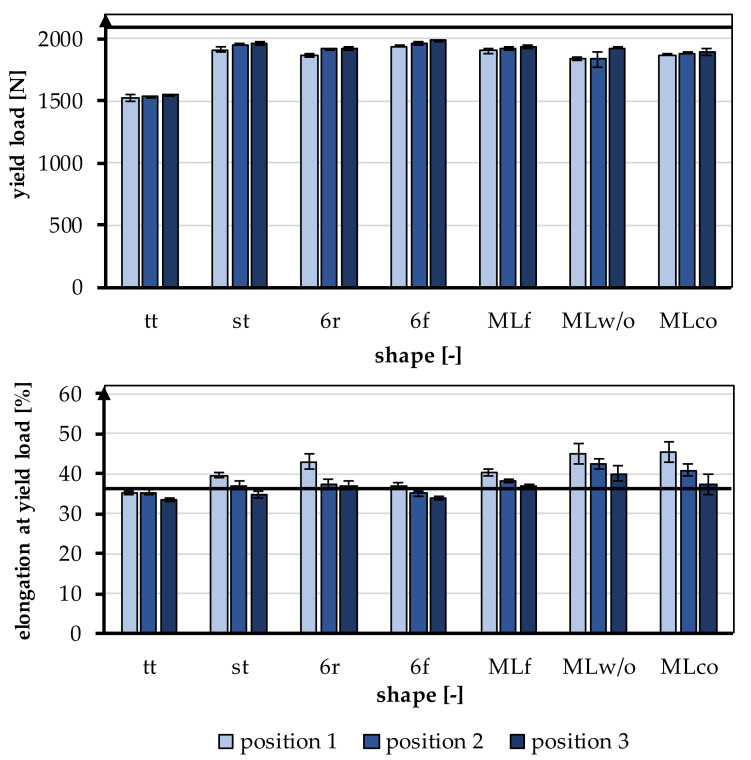
Yield load and elongation at yield load of the braided scaffolds in comparison to mechanical properties of the native ACL [11].

The behavior of the braids in the linear region of the tensile tests is relevant for physiological use as a cruciate ligament and was therefore focused on the evaluation. The linear region of the test is defined by the yield load and the elongation at the yield point. The yield load and the elongation at the yield point are shown in Figure 5. The yield load was 1858.63 ± 138.58 N on average, and the elongation at the yield point was 38.3 ± 3.64%. A comparison of the mechanical properties, yield load, and elongation at yield load with the properties of the human ACL is given in Table 4. A more detailed comparison is found in Section 4.

**Figure 6:**
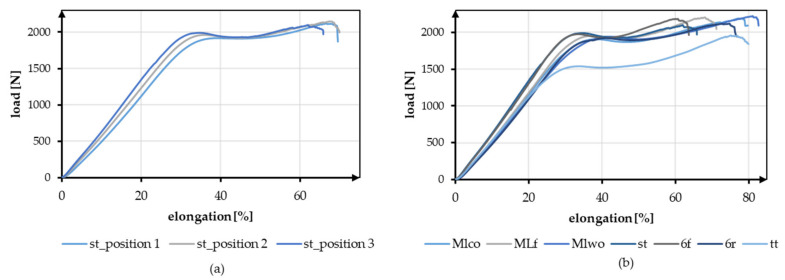
Exemplary tensile test data (**a**) Comparison of braid st at braid positions 1–3, (**b**) Comparison of all geometries at braiding position 3.

The effect of the different braiding positions and the different geometries is shown in Figure 6. Figure 6a shows exemplary tensile test data of braid st at the three braiding positions. Figure 6b shows the exemplary load-elongation curves for all geometries at position 3.

#### 3.1.1. Yield Load

The yield load was significantly influenced by the shape of the braid (*p* < 0.001) and the braiding position (*p* < 0.001). A higher braiding position resulted in a significantly higher yield load. The minimum tensile load requirement of 1700 N was achieved by all braids except tt. The occurring yield load of all braiding shapes was ranked between 1522.23 ± 27.86 N (tt, position 1) and 1986.53 ± 8.37 N (6f, Position 3) (Figure 6).

The influence of different braiding shapes on the yield load was considered in the study. The scale-up of the braids from tt to st showed significant differences in tensile loads (*p* < 0.001). The st had on average a higher tensile load compared to the tt.

The comparison between flat and round braids was carried out using MLco against MLf, and 6r against 6f. The variation of the braid shape from round and flat led to significant differences in yield load (*p* < 0.001). The flat braids attained a higher yield load than the round braids. The MLf braid achieved the highest value with an average of 1936.35 ± 9.52 N in position 3, and the MLco exhibited 1892.77 ± 24.69 N in position 3. The 6f also attained a higher yield load with an average of 1986.53 ± 8.37 N in position 3 than the round 6r, which reached 1920.88 ± 16.47 N in position 3.

The different connections of the individual strands in 6r and st also affected the tensile load. The central connection resulted in a significantly higher yield load compared with the connection over the edge (*p* < 0.001). The 6r, therefore, achieved the lower value with an average of 1920.88 ± 16.47 N in position 3, and the st attained 1960.52 ± 16.65 N in position 3. The connection of the layers in the comparison MLco and MLwo has no significant effect (*p* = 0.15).

#### 3.1.2. Elongation at Yield Point

The elongation at the yield point was significantly influenced by the braid shape (*p* < 0.001) and the braiding position (*p* < 0.001). A higher braiding position resulted in significantly lower elongation at yield load. The elongations were slightly higher than the physiologically occurring elongations in a ligament (up to 37%). The elongations ranked between 33.56 ± 0.48% (tt, position 3) and 45.42 ± 2.68% (MLco, position 1) (Figure 6).

The influence of different structural adjustments on the elongation at the yield point was considered in the study. The scale-up of the braids from tt to st showed significant differences in the elongations that occur (*p* < 0.001). The st shape attained higher elongations on average than the tt shape.

The comparison between flat and round braids was carried out using MLco against MLf, and 6r against 6f. The flat braids achieved significantly lower elongations than the round braids (*p* < 0.001). The MLf braid reached the highest value of 40.39 ± 0.81% in position 1, and MLco attained 45.42 ± 2.68% in position 1. The 6f achieved 36.97 ± 0.65% in position 1, while 6r exhibited 42.88 ± 1.87% in position 1. The different connections of the individual strands in 6r and st also affected the elongation at yield load. The elongation in the braids with a central connection was lower than in the braids with a connection over the margin (*p* < 0.001). The 6r, therefore, achieved the higher value with an average of 42.88 ± 1.87% in position 1, and the st attained 39.57 ± 0.64% in position 1. The connection of the layers in the comparison MLco and MLwo also had no significant effect (*p* = 0.17).

#### 3.1.3. Stiffness—Linear Region

The stiffness is normalized to the length of the tensile specimen. During the tensile tests, the stiffness in the linear region was determined between 200 N and 1000 N, which describes the course of the tensile curve in the linear region. On average, the stiffness was 59.13 ± 3.93 N/mm. The stiffness is significantly influenced by the braid shape (*p* < 0.001) and by the braid position (*p* < 0.001). The stiffness increased with a higher braiding point. The highest stiffness was found in the 6f braid in position 3 with 66.41 ± 0.74 N/mm, and the lowest stiffness was measured in the MLwo braid in position 1 with 52.21 ± 0.89 N/mm.

The influence of the different braiding shapes on the stiffness was considered in the study. The scale-up of the braids from tt to st showed significant differences in the resulting stiffness (*p* < 0.001). The st braids (65.45 ± 1.53 N/mm in position 3) were stiffer than the tt braids (59.44 ± 0.91 N/mm in position 3).

The comparison between flat and round braids was carried out using MLco against MLf and 6r against 6f. The flat braids achieved significantly higher stiffness than the round braids (*p* < 0.001). The MLf braid attained the highest value in position 3 with 62.3 ± 0.46 N/mm and is thus stiffer than the MLco braid in position 3 with 60.23 ± 0.61 N/mm. The 6f braid exhibited 66.41 ± 0.74 N/mm in position 3, and the 6r braid had the lowest stiffness with 60.13 ± 1.58 N/mm in position 3.

The different connections of the individual strands in 6r and st also affected the stiffness. The central connection was significantly higher in stiffness compared to the marginconnected braids (*p* < 0.001). Therefore, the stiffness of st was higher with 65.45 ± 1.53 N/mm in position 3 than the stiffness attained by a connection over the margin (6r) with 60.13 ± 1.58 N/mm. The connection of the layers in the comparison MLco and MLwo had no significant effect (*p* = 0.15).

#### 3.1.4. Stiffness—Toe Region

During tensile tests, the stiffness of the toe region was determined between 10 N and 40 N, in addition to the stiffness described above. The average stiffness in the toe region was 32.03 ± 5.59 N/mm. Comparable to the stiffness described above, the stiffness in the toe region was significantly influenced by the braid shape (*p* < 0.001) and the braid position (*p* < 0.001). The stiffness in the toe region increased with a higher braid point. The 6f braid in position 3 had the highest stiffness with 43.1 ± 0.64 N/mm, and the 6r braid in position 1 had the lowest stiffness with 23.6 ± 1.15 N/mm.

The influence of different braiding shapes on stiffness was considered in the study. The scale-up of the braids from tt to st showed significant differences in the occurring stiffness (*p* < 0.001). The st braids (39.88 ± 3.27 N/mm in position 3) were stiffer than the tt braids (34.22 ± 1.6 N/mm in position 3).

The comparison between flat and round braids was carried out using MLco against MLf and 6r against 6f. An influence of the shape on the stiffness could not be seen. The MLf braid and the MLco braid did not differ significantly (*p* = 0.35), while 6r and 6f differed significantly (*p* < 0.001). The 6r reached a maximum value of 30.86 ± 1.55 N/mm in position 3, while 6f reached 43.1 ± 0.64 N/mm. In comparison to that, the MLco braid reached 33.80 ± 1.47 N/mm in position 3, while MLf reached 32.51 ± 1.05 N/mm.

The different connections of the individual strands in 6r and st also affected the stiffness. The central connection was significantly higher than the connection over the edge (*p* < 0.001). The connection of the plies in the comparison MLco and MLwo (33.63 ± 1.73 N/mm in position 3) had no significant effect (*p* = 0.2).

#### 3.1.5. Influence of the Gauge Length

All specimens were tested with a gauge length of 100 mm according to the standard NF S 94-167-2. To allow a better comparison to the physiological properties of the native cruciate ligament, specimens of the braid geometry tt were tested with a 40 mm gauge length. The mechanical properties are shown in Table 5.

**Figure 7:**
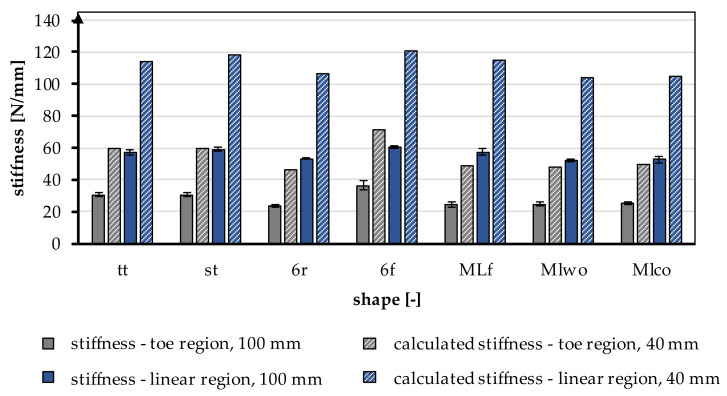
Stiffness in the toe region and linear region of different braiding shapes was tested with a gauge length of 100 mm, calculated stiffness for a gauge length of 40 mm according to the native length of an ACL, conversion factor based on Table 5.

The stiffness was increased by a factor of 2 in the linear region and by a factor of 1.96 in the toe region by shortening it to 40 mm. In Figure 7, the experimentally calculated factor is applied to all braiding shapes. The shortening of the braid also significantly influenced the resulting yield load and measured elongations. The loads and the elongations increased with a shorter specimen length.

### 3.2. Scaffold Morphology

In Figure 2, the investigated 3D-braided scaffolds are depicted. The cross-section is shown in the Matlab visualization, the top view using light microscopy, and the 3D structure illustrated by µCT scans. Light microscopy pictures and µCT scans are braids manufactured in position 1.

#### 3.2.1. Porosity and Pores Size Distribution of 3D-Braided Scaffolds

**Figure 8:**
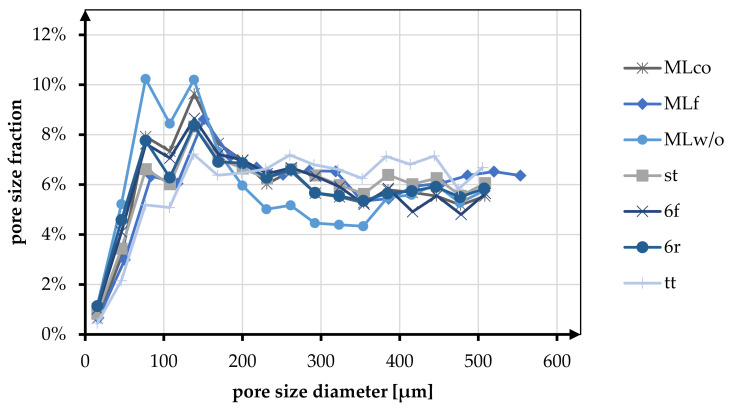
Fraction of different pore sizes in relation to the braiding shapes in position 3.

Pore size and porosity are crucial variables for the suitability of tissue engineering scaffolds. In Table 6 and Figure 8, the porosity and pore size distribution for all braid geometries are shown at braiding position 3.

At position 3, the average porosity of all braid geometries was 85.92%. The change in braid position from position 1 to position 3 showed a tendency to increase porosity. For the MLco braid, the porosity increased from 80.46% to 85.85%. The same tendency was observed, for instance, for the braids 6f (position 1: 86.62%, position 3: 88.23%) and st (position 1: 82.62%, position 3: 84.75%).

In the following, the morphological characteristics are only considered for position 3, being the most similar to the anterior cruciate ligament in terms of mechanical properties (low breaking elongation and high stiffness). The highest porosity is shown in the tt braid with 91.32%, and the lowest porosity is found in the MLw/o braid with 81.55%. Comparing round and flat braid, there was no difference between MLco (85.85%) and MLf (85.17%). In contrast, the comparison of 6f and 6r showed an increase in porosity for the flat geometry. Comparing the braid geometries with different connection mechanisms (center connected and margin connected), no difference in porosity was observed. The braids 6r and st exhibited porosity of 84.59% and 84.75%, respectively. For the multilayer braids, the connection of the layers showed an increase in porosity from 81.55% (MLw/o) to 85.85% (MLco).

The pore size of the braids was analyzed in the pore size up to 500 µm. Here the pore sizes below and above 245 µm were distinguished based on the histogram distribution. The tt braid had the highest percentage of pores in the range above 245 µm with 60.44%. MLw/o had the highest percentage of pores smaller than 245 µm with 53.55%. On average, the proportion of pores below 245 µm for all braid geometries was 42.82%, and the proportion larger than 245 µm was 52.90%.

The pore size distribution of all braids was similar, having a maximum below 200 µm and a uniform distribution in the range of 200–500 µm. Exceptions to this were braid tt, where no clear maximum was identified, and braid MLw/o, which showed two maxima in the pore size range below 200 µm.

In the comparison related to the influence of the braid geometries round and flat, an equal distribution of the pores is shown for the braids 6r and 6f. Both in terms of the fractions above and below 245 µm (<245 µm 49.11% (6f), 48.18% (6r); >245 µm 50.89% (6f), 51.82% (6r)) and in terms of the distribution up to 500 µm. MLco and MLf exhibited a slight shift toward larger pores for the flat braid in comparison. The proportion larger than 245 µm is 55.12% compared to 50.71% for MLco. The same tendency could be seen in the histogram. The pore size distribution of MLco and MLf up to 500 µm was similar, and only MLf showed a shift of the maxima to slightly larger pore sizes.

As with the comparison regarding porosity, there was no influence on the pore size distribution due to the connection of the braids (margin or center connected). st and 6r showed an identical pore size distribution with an identical maximum at 138 µm. The comparison with respect to multilayer braids with and without interconnection exhibited a higher pore size fraction below 245 µm for MLw/o (53.55% (MLw/o), 49.29% (MLco)).

#### 3.2.2. Cross-Section of the 3D-Braided Scaffolds

The cross-sectional area of the braided scaffolds was compared with the cross-sectional area of the native ACL. The minimum cross-sectional area is given at 44 mm^2^ [10]. The area of the minimum drill holes in established surgery methods is given at 7 mm, corresponding to 38.48 mm^2^ [9]. The test specimens all had a cross-sectional area that was less than 38.48 mm^2^ while applying 2 N tension. The values of the measured cross-sectional areas differed only marginally between the braiding positions. Therefore, only the mean values of the three positions were considered.

The cross-sectional area under tension ranged between 9.55 ± 0.35 mm^2^ (MLf) and 15.97 ± 1.23 mm^2^ (tt). By comparing the multilayer braids, the connection of the braiding layers had no influence on the cross-sectional area, with 11.98 ± 0,9 mm^2^ (MLwo) and 11.47 ± 0.78 mm^2^ (MLco), respectively. The braids with a center-connected structure showed a slightly lower cross-sectional area (11.59 ± 0.72 mm^2^ (st), 14.07 ± 3.18 mm^2^ (6r)).

Comparing the flat and round braids, specifically MLco and MLf, as well as 6r and 6f, smaller cross-sectional areas were measured for the flat braids.

## 4. Discussion

The main objective of this study was to investigate a novel biodegradable 3D-braided ACL scaffold for ligament reconstruction. In specific, the focus was on the large geometric freedom of the 3D hexagonal braiding technology as well as the influence of the braid height on the mechanical properties, pore size, and porosity. Using the 3D hexagonal braiding technique, seven different braids were developed and produced at three different braiding positions. The developed designs were divided into three groups (1) round and flat braids, (2) multilayer braids with and without connection, and (3) multi-tube braids with different connection mechanisms.

In contrast to circular braiding and overbraiding, 3D braiding offers tremendous freedom in the geometry of the braid.

Mengsteab et al. used a row and column braiding machine and described the possibility of producing braids with rectangular and square cross-sections [45]. Cooper et al. utilized a similar braiding machine to fabricate rectangular braids. In a second process step using a 3D-braiding machine with a circular machine bed, a combination of a braid with rectangular and circular cross-sections could be produced [43]. In the present study, the versatility of braid geometries using 3D hexagonal braiding technology was demonstrated. Due to the possibility of producing complex round braids as well as rectangular shapes on a single machine, the 3D hexagonal braiding technique is more versatile compared to row and column braiding machines. In contrast to the study of Cooper et al., one process step is spared to produce a scaffold with round and rectangular geometry.

The braids studied had predominantly round cross-sections, as these were adapted more closely to the round bone tunnel, resulting in uniform force application in the braid and improved contact of the scaffold with the surrounding bone tissue. The variability of the 3D hexagonal braids also offers the possibility of developing new fixation techniques. For instance, metal anchoring plates can be integrated directly into the braiding process, thus simplifying the process chain. Furthermore, it is possible to split the braid into a bifurcation or several strands without losing the braid’s structural integrity. This enables further fixation techniques.

The maximum tensile load of the human ACL depends on numerous factors. These include the patient’s age, sex, and physical activity. The maximum tensile load varies from 734–1730 N to 2160 N, depending on the respective study. The load during daily activity ranges from 169 N (normal walking) to 445 N (descending stairs) [10,12]. The braided scaffolds achieved tensile loads between 1522 N and 1986 N. Thus, all braids except braid tt reached the maximal tensile loads of the native ACL of a young human. Due to the tissue engineering approach, which requires a suitable healing capacity, the mechanical properties of the ACL of a young human were taken as a reference. The elongation of the natural ACL is 37% at maximum load. Comparing this with the scaffolds produced, these were in a similar elongation range at 33.56% and 45.42%, respectively. The elongation of the braids can be further reduced while increasing the stiffness by adjusting the braiding parameters. For example, the braiding angle can be further decreased, corresponding to a larger distance between the braiding point and the machine bed. It should be noted that the braid shape has less structural integrity when the braiding angle is very small. Therefore, another possibility is to integrate inlay yarns into the 3D braid. The effect size of this variation should be investigated in further studies. The average absolute stiffness of the braids with a length of 100 mm was 59.12 N/mm. Theoretically, the stiffness of the braids increased on average to 118.24 N/mm by shortening the scaffolds to the length of the native ACL of approximately 40 mm. The achieved stiffness was in the range of the stiffness of the native ACL with 75–220 N/mm for the age group above 40 years and below the stiffness of the age group below 40 years with 182–242 N/mm [10,11,13]. The stiffness of the 3D-braided scaffolds could be easily increased by increasing the number of fibers. The diameter of the braids being far smaller than the required bone tunnel diameter, there is still ample room for increasing the number of fibers without exceeding the native ACL geometry.

The elongation in the linear region was significantly affected by braid geometry and braid position. A higher braiding position led to a lower elongation at yield strength due to the more linear orientation of the fibers. The absolute stiffness was also influenced by braid geometry and braid position. A higher braid position resulted in a higher stiffness [51]. Jedda et al. and Turki et al. have also demonstrated that a more parallel alignment of the fibers at a lower braid angle results in higher stiffness [52,53]. The yield load was also significantly influenced by the braiding geometry and the braiding position. A higher braiding position, corresponding to a lower braiding angle, resulted in a higher yield load. The same influences of angle/braid position were shown by Laurent et al. and Mengsteab et al. for ACL scaffolds based on round braids and quadrangular 3D braids, respectively [41,45].

Based on the literature, a porosity of 50–80% is desirable for tissue engineering scaffolds to allow sufficient in vivo tissue ingrowth [54]. As Cooper et al. have pointed out, 3D braids also provide suitable conditions for the transport of oxygen and nutrients through the interconnected pore structure.

All developed 3D braids had a porosity of at least 81%, so a sufficient porosity for in vivo tissue ingrowth was achieved. In ACL reconstructions, the ingrowth of bone tissue for anchorage in the bone tunnels, as well as of tenocytes for remodeling the ligament structure, is an important factor. Therefore, the adaptability of the scaffold structure to the requirements of different tissues is crucial. For bone tissue, a minimum pore size of 150 µm is specified, and for soft tissue, 200–250 µm [55,56,57,58]. The presented 3D braids had approximately 50% in the range of analyzed pores (up to 500 µm), which were below 250 µm and thus formed the target range, respectively, the minimum for the previously mentioned tissues. By changing the braiding parameters, especially the height of the braiding point, lower porosity and a shift of the pore size distribution toward smaller pores can be achieved. This is in agreement with the results of Cooper et al.

To adapt the scaffold structure to different tissues, as in Cooper et al., as well as to occurring mechanical loads in the bone and ligament area, a modular structure of the scaffold with different braiding parameters can be investigated in further studies. Denser braids can be chosen for the section in the bone to withstand the frictional forces at the edges of the drill channel and to fulfill the pore size requirement for the bone tissue. In the ligament section, braids with a low braiding angle can be used for low elongation and high stiffness while maintaining high porosity and providing a structure for growth guidance for native tissue.

## 5. Conclusions

Tissue engineering for ligament replacement is a promising approach to overcome the limitations of existing solutions, which have limitations in availability and are associated with donor site morbidity, such as autologous implants, or show poor results for fatigue strength, such as synthetic implants.

In this study, we identified significant influencing parameters to produce a fibrous, tissue-engineered ligament replacement using 3D-braiding technology. It was shown that melt-spun PCL fibers are a suitable material for a load-bearing anterior cruciate ligament implant. The mechanical properties of the braid, especially the stiffness, were significantly influenced by the braiding angle. In summary, braid position three can be considered a promising configuration in terms of mechanical properties, higher stiffness, higher yield load, and lower elongation in combination with a braid with round geometry, e.g., center-connected six-fold tubular braid (st) for an ACL scaffold. The geometrical freedom of the 3D-braiding technology can be used to extend the area of applications to other ligament structures.

Future studies will focus on increasing the scaffold’s stiffness and investigating long-term mechanical behavior under cyclic load in a physiological range. In vitro characterization of the cellular response and interaction with the braided tissue-engineered ligament scaffold will be investigated.

## Figures and Tables

**Table 1:** Properties of the melt-spun PCL fibers used for 3D-braiding of the scaffolds.

Properties	Value
Fineness [dtex]	378.15 ± 0.74
Material [-]	PCL
Tenacity [cN/tex]	40.45 ± 1.88
Cross-sectional shape [-]	round

**Table 2:** Comparison of the different braiding geometries produced by 3D-braiding.

Comparison	Included Braiding Geometries
Influence of flat and round braiding geometry	MLco vs. MLf 6r vs. 6f
Influence of the connection of multilayer braiding geometries	MLco vs. MLw/o
Influence of the connection mechanism (margin connected and center connected)	st vs. 6r

**Table 3:** Results of tensile tests of all seven braid shapes at braiding positions 1–3.

Shape	Center Connected Threefold Tubular Braid (tt)			Center Connected Sixfold Tubular Braid (st)			Margin Connected Sixfold Tubular Braid (6r)			Margin Connected Sixfold Flat Braid (6f)			Multilayer Flat Connected (MLf)			Multilayer without Connection (MLw/o)			Multilayer Connected (MLco)		
Position	1	2	3	1	2	3	1	2	3	1	2	3	1	2	3	1	2	3	1	2	3
**ultimate strength F_max_ [N]**	1841.7 ± 133.3	1944.2 ± 71.6	1959.9 ± 18.3	2128.4 ± 23.1	2122.3 ± 20.9	2078.1 ± 20.8	2157.8 ± 35.8	2145.0 ± 12.0	2098.2 ± 79.5	2194.0 ± 22.2	2175.5 ± 25.5	2161.9 ± 20.2	2193.7 ± 42.0	2189.3 ± 22.7	2181.2 ± 19.9	2220.5 ± 25.5	2132.3 ± 111.3	2142.3 ± 42.1	2283.6 ± 44.8	2228.5 ± 16.2	2217.8 ± 22.8
**ɛ at Fmax [%]**	72.2 ± 8.3	77.4 ± 3.8	76.1 ± 1.7	69.5 ± 2.7	69.3 ± 1.07	63.1 ± 2.5	81.0 ± 2.2	70.4 ± 2.2	67.7 ± 6.6	66.9 ± 1.4	65.4 ± 1.4	60.7 ± 1.4	73.6 ± 3.4	68.7 ± 2.7	66.8 ± 1.0	88.8 ± 1.6	80.5 ± 5.1	76.3 ± 3.8	91.5 ± 4.4	81.4 ± 1.9	77.0 ± 0.7
**ɛ at break [%]**	75.4 ± 9.1	82.8 ± 3.5	81.6 ± 2.8	70.92 ± 2.6	72.49 ± 1.5	66.2 ± 2.5	82.4 ± 2.1	73.0 ± 1.0	70.5 ± 4.5	69.1 ± 1.1	66.9 ± 1.6	64.2 ± 0.6	77.05 ± 3.1	73.50 ± 2	70.4 ± 0.53	90.25 ± 1.3	82.71 ± 4.23	77.93 ± 3.4	92.9 ± 4.2	83.5 ± 1.0	79.3 ± 1.0
**yield load [N]**	1522.2 ± 27.9	1534.9 ± 8.7	1550.0 ± 7.0	1909.9 ± 21.6	1954.9 ± 6.9	1960.5 ± 16.7	1868.2 ± 10.1	1915.2 ± 11.3	1920.9 ± 16.5	1938.8 ± 4.7	1958.5 ± 16.6	1986.5 ± 8.4	1902.6 ± 15.9	1926.4 ± 12.6	1936.4 ± 9.5	1837.3 ± 14.3	1833.8 ± 59.7	1925.9 ± 10.5	1870.4 ± 7.9	1885.2 ± 9.7	1892.8 ± 24.7
**elongation at yield load [%]**	35.2 ± 0.6	35.3 ± 0.7	33.6 ± 0.5	39.6 ± 0.6	37.1 ± 1.0	34.8 ± 0.8	42.9 ± 1.9	37.2 ± 1.3	37.0 ± 1.0	37.0 ± 0.7	35.1 ± 0.6	33.9 ± 0.4	40.4 ± 0.8	38.1 ± 0.6	36.8 ± 0.6	44.9 ± 2.7	42.3 ± 1.2	40.0 ± 1.9	45.4 ± 2.7	40.8 ± 1.5	37.3 ± 2.5
**Stiffness—toe region [N/mm]**	30.5 ± 1.5	32.1 ± 1.2	34.2 ± 1.6	30.6 ± 1.3	38.8 ± 1.8	39.9 ± 3.3	23.6 ± 1.2	29.0 ± 1.9	30.9 ± 1.6	36.5 ± 3.2	40.1 ± 1.6	43.1 ± 0.6	24.7 ± 1.6	30.1 ± 3.1	32.5 ± 1.1	24.6 ± 1.2	28.5 ± 2.5	33.6 ± 1.7	25.4 ± 0.7	30.2 ± 1.9	33.8 ± 1.5
**stiffness—linear region [N/mm]**	56.9 ± 1.6	56.9 ± 0.7	59.4 ± 0.9	59.1 ± 1.4	61.4 ± 1.3	65.5 ± 1.5	53.3 ± 0.6	60.5 ± 1.2	60.1 ± 1.6	60.6 ± 0.7	64.1 ± 1.2	66.4 ± 0.7	57.4 ± 2.1	60.4 ± 0.7	62.3 ± 0.5	52.2 ± 0.9	55.9 ± 1.4	59.4 ± 1.2	52.6 ± 1.9	57.0 ± 1.2	60.2 ± 0.6

**Table 4:** Comparison of the different 3D braided geometries at braiding position 3 with the properties of the human ACL [10,12].

Shape	Human ACL	Center Connected Threefold Tubular Braid (tt)	Center Connected Sixfold Tubular Braid (st)	Margin Connected Sixfold Tubular Braid (6r)	Margin Connected Sixfold Flat Braid (6f)	Multilayer Flat Connected (MLf)	Multilayer Without Connection (MLw/o)	Multilayer Connected (MLco)
Position	-	3	3	3	3	3	3	3
Yield load [N]	734 − 2160	1550.0 ± 7.0	1960.5 ± 16.7	1920.9 ± 16.5	1986.5 ± 8.4	1936.4 ± 9.5	1925.9 ± 10.5	1892.8 ± 24.7
Elongation at yield load [%]	37	33.6 ± 0.5	34.8 ± 0.8	37.0 ± 1.0	33.9 ± 0.4	36.8 ± 0.6	40.0 ± 1.9	37.3 ± 2.5
Porosity [%]	-	91.32	84.75	84.59	88.23	85.17	81.55	85.85

**Table 5:** Comparison of the stiffness in the toe region and linear region of the braid shape tt in position 1 with gauge lengths 40 mm and 100 mm, calculation of the conversion factor used in Figure 7. Further information is displayed in Table A1 in Appendix A.

	Parameter of a tt Braid in Position 1		Conversion Factor [-]
Length of braid [mm]	40	100	N/A
Stiffness–linear region [N/mm]	114.98 ± 8	56.88 ± 0.66	2.0
Stiffness–toe region [N/mm]	62.78 ± 2.93	32.1 ± 1.2	1.96

**Table 6:** Porosity and pore size fraction of different braid shapes in position 3 in the range of relevant pore sizes < 250 µm for soft tissue ingrowth [43].

Braid Shape (-)	Fraction of Pores < 245 µm (%)	Fraction of Pores > 245 µm (%)	Porosity (%)
MLco	49.29	50.71	85.85
MLf	44.88	55.12	85.17
MLw/o	53.55	46.45	81.55
st	45.14	54.86	84.75
6f	49.11	50.89	88.23
6r	48.18	51.82	84.59
tt	39.56	60.44	91.32

## Data Availability

Not Applicable.

## Appendix A

**Table A1:** Results of tensile tests of tt braid with a different gauge length.

Shape	Center-Connected Three-Fold Tubular Braid (tt)	
Position	1	1
Gauge length (mm)	100	40
Ultimate load F_max_ (N)	1841.73 ± 133.3	2083.79 ± 57.22
ɛ at F_max_ (%)	72.15 ± 8.31	106.67 ± 6.69
ɛ at break (%)	75.39 ± 9.09	116.51 ± 4.14
Yield load (N)	1522.23 ± 27.86	1562.52 ± 8.52
Maximum elongation at yield Load (%)	35.19 ± 0.56	47.59 ± 2.87
Stiffness—toe region (N/mm)	30.52 ± 1.45	62.78 ± 2.93
Stiffness—linear region (N/mm)	56.89 ± 1.56	114.98 ± 8
